# Profiling the Kidney Before the Incision: CT-Derived Signatures Steering Reconstructive Strategy After Off-Clamp Minimally Invasive Partial Nephrectomy

**DOI:** 10.3390/cancers17193236

**Published:** 2025-10-05

**Authors:** Umberto Anceschi, Antonio Tufano, Davide Vitale, Francesco Prata, Rocco Simone Flammia, Federico Cappelli, Leonardo Teodoli, Claudio Trobiani, Giulio Eugenio Vallati, Antonio Minore, Salvatore Basile, Riccardo Mastroianni, Aldo Brassetti, Gabriele Tuderti, Maddalena Iori, Giuseppe Spadaro, Mariaconsiglia Ferriero, Alfredo Maria Bove, Elva Vergantino, Eliodoro Faiella, Aldo Di Blasi, Rocco Papalia, Giuseppe Simone

**Affiliations:** 1 Department of Urology, Uro-oncology Program, IRCCS “Regina Elena” National Cancer Institute, 00144 Rome, Italy; 2 Department of Urology, San Carlo di Nancy Hospital GVM Care and Research, 00165 Rome, Italy; 3 Department of Radiology, IRCCS “Regina Elena” National Cancer Institute, 00144 Rome, Italy; 4 Department of Urology, Fondazione Policlinico Universitario Campus Bio-Medico, 00128 Rome, Italy; 5 Department of Radiology, Fondazione Policlinico Universitario Campus Bio-Medico, 00128 Rome, Italy; 6 Department of Radiology, San Giovanni Evangelista Hospital (ASL RM 5)—Tivoli, 00019 Rome, Italy

**Keywords:** minimally invasive, partial nephrectomy, robotic, predictors, radiologic, CT scan, off-clamp, sutureless

## Abstract

**Introduction:** In minimally invasive, off-clamp partial nephrectomy (ocMIPN), the reconstructive strategy profoundly influences functional outcomes. Traditional nephrometry scores aid preoperative planning but do not directly inform the choice of closure technique. This dual-institutional study aimed primarily to identify preoperative CT-derived parameters predictive of renorrhaphy versus a sutureless approach, and secondarily to compare perioperative and functional outcomes between these techniques. **Methods:** We retrospectively analyzed 201 consecutive ocMIPN cases performed using a standardized off-clamp technique by two experienced surgical teams across robotic platforms and conventional laparoscopy. Preoperative CT scans were centrally reviewed to quantify morphometric features, including contact surface area (CSA), tumor radius, and Gerota’s fascia thickness. Univariable and multivariable logistic regression models—one restricted to radiologic variables and one expanded with RENAL score terms—were generated to identify independent predictors. Perioperative outcomes, renal functional metrics, and Trifecta rates were compared between cohorts. **Results:** Among the 201 patients, 101 (50.2%) underwent sutureless reconstruction and 100 (49.8%) renorrhaphy. Cohorts were comparable at baseline except for tumor size (3.1 vs. 3.6 cm; *p* = 0.04). In multivariable analysis, CSA > 15 cm^2^ (OR 3.93; 95% CI 1.26–12.26; *p* = 0.02) and tumor radius (OR 1.14 per mm; 95% CI 1.01–1.29; *p* = 0.04) consistently predicted renorrhaphy, while Gerota’s fascia < 10 mm emerged as significant only in the expanded specification (OR 0.08; 95% CI 0.01–0.70; *p* = 0.02). Integration with RENAL improved predictive performance (ΔAUC 0.06; NRI 0.14; IDI 0.07), and the final model demonstrated strong discrimination (AUC 0.81) with satisfactory calibration. Perioperative outcomes, postoperative renal function, and Trifecta achievement were similar between groups (all *p* ≥ 0.21). **Conclusions:** A concise set of CT-derived morphologic markers—CSA, tumor radius, and perinephric fascia thickness—anticipated reconstructive strategy in ocMIPN and augmented the discriminatory power of RENAL nephrometry. When anatomy was favorable, sutureless repair was not associated with statistically significant differences in perioperative safety or renal function, although the study was not powered for formal equivalence testing. These findings support the integration of radiologic markers into preoperative planning frameworks for nephron-sparing surgery.

## Simple Summary

This study examined how kidneys are reconstructed after minimally invasive partial nephrectomy performed without vascular clamping. Conventional scoring systems assist in surgical planning but do not inform the choice of whether renorrhaphy is required. In a consecutive dual-institution cohort of 201 patients treated with robotic and laparoscopic approaches, preoperative CT scans were systematically analyzed to quantify tumor–parenchyma contact area, tumor radius, and perinephric fascia thickness. These morphologic markers were evaluated for their ability to anticipate the need for suturing versus a sutureless repair. Surgical outcomes, postoperative renal function, and composite success rates were compared between techniques. During follow-up, no local recurrences and only a limited number of distant events were observed; however, the study was primarily designed to address reconstructive strategy rather than oncologic endpoints. The findings provide practical, imaging-based guidance that may refine operative planning in contemporary kidney-sparing surgery.

## 1. Introduction

The widespread integration of robotic platforms has redefined the operative landscape of nephron-sparing surgery, with minimally invasive partial nephrectomy (MIPN) now representing the standard approach for the management of localized renal tumors in many referral centers [1,2,3]. This evolution has refined both the excisional and reconstructive phases of the procedure, allowing for increasingly individualized strategies in the treatment of anatomically complex lesions [4,5,6]. Within this framework, the reconstructive phase following tumor excision plays a pivotal role in balancing oncologic control with preservation of renal function [7,8]. The choice of technique to achieve hemostasis and restore parenchymal integrity is not merely a technical nuance, but a determinant of surgical complexity, operative time, and potential impact on postoperative outcomes [9,10]. Yet, despite its clinical relevance, this phase remains variably approached across centers, often relying more on intraoperative judgment than on objective preoperative indicators.

To support surgical planning and standardize preoperative assessment, nephrometric scoring systems, such as the R.E.N.A.L. and PADUA scores, are routinely applied to estimate tumor complexity and predict perioperative complications [11,12]. These tools have become essential components of preoperative stratification and are widely used in clinical decision-making and outcome reporting [13,14]. Although these systems offer a structured appraisal of anatomical difficulty, they were not specifically designed to inform intraoperative decisions concerning the reconstructive phase following tumor enucleation. In most cases, renorrhaphy remains the conventional approach to secure hemostasis and preserve parenchymal integrity. However, alternative strategies—including sutureless techniques based on selective coagulation—have emerged in selected scenarios, supported by the availability of advanced energy devices and adjunctive hemostatic agents [15,16,17,18].

Despite increasing interest in the optimization of renal reconstruction, the extent to which preoperative imaging can predict the surgical approach to hemostasis remains poorly defined [19]. This dual-institutional study aimed to investigate whether specific CT-derived features can predict the surgical decision to perform renorrhaphy versus pursue a sutureless reconstruction during off-clamp minimally invasive tumor enucleation, thereby enabling a more informed and morphology-driven approach to parenchymal repair.

## 2. Materials and Methods

### 2.1. Patient Selection

Between January 2019 and July 2025, we retrospectively reviewed 201 consecutive patients who underwent off-clamp partial nephrectomy (ocMIPN) for a contrast-enhancing unilateral renal mass at two academic institutions. All procedures—whether laparoscopic or robot-assisted—were performed using a standardized off-clamp technique by two experienced minimally invasive surgeons beyond their respective learning curves (defined as >50 prior ocMIPN cases). Robotic procedures were conducted with the da Vinci Xi Surgical System (Intuitive Surgical, Sunnyvale, CA, USA) at the IRCCS Regina Elena National Cancer Institute and with the Hugo RAS System (Medtronic, Dublin, Ireland) at Campus Bio-Medico University Hospital. Laparoscopic cases were performed by the same surgeons, depending on robotic platform availability and case-specific considerations.

Across centers, reconstructive choice followed a predefined rule: sutureless closure was considered only when the enucleation bed was dry and hemostasis was achievable with energy devices without parenchymal compression; otherwise, a single-layer cortical renorrhaphy was performed. Energy sources were limited to monopolar scissors and bipolar forceps. Adjunctive topical hemostatic agents were not used systematically, and no parenchymal bolsters, sealants, or buttressing materials were employed. Hilar clamping was not used in any case.

Inclusion criteria comprised the availability of complete perioperative and follow-up data, as well as a dedicated preoperative renal protocol CT scan suitable for central radiologic review. Exclusion criteria included multifocal or bilateral tumors, procedures conducted during the surgeon’s learning phase, and incomplete imaging or clinical datasets. Institutional Review Board approval was granted by both centers (IFO #RM-IFO-23-0072; CBM #CAMPUS-24-0115). For patients treated before 2024, a waiver of consent was granted in accordance with national regulations on retrospective research, while for cases accrued thereafter, written informed consent for inclusion in the research registry was systematically obtained.

**Figure 1:**
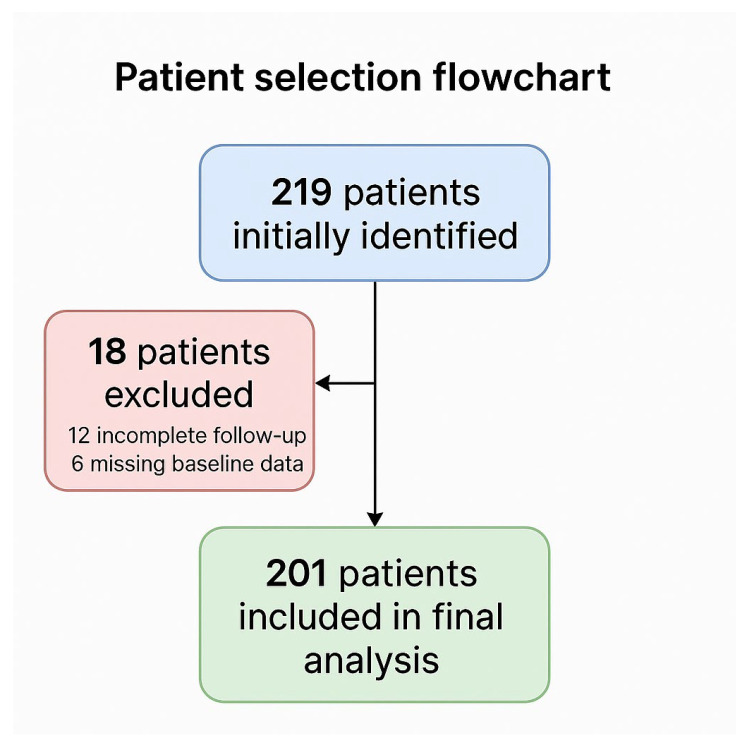
Patient selection flowchart according to STROBE recommendations.

Across the two participating institutions, 219 patients who underwent off-clamp minimally invasive partial nephrectomy were initially identified. Eighteen were excluded: twelve due to incomplete follow-up and six for missing baseline data. Thus, 201 patients fulfilled all eligibility criteria and were retained for the present analysis. Patient selection and attrition are summarized in a flow chart (Figure 1), in accordance with STROBE recommendations for observational studies.

### 2.2. Radiologic Assessment

Preoperative renal protocol CT scans were independently reviewed by four experienced radiologists (V.D., V.E., F.E., and V.G.), each affiliated with one of the participating centers. Prior to study initiation, the radiologists jointly defined a set of standardized morphometric and qualitative parameters to be assessed in all cases.

The following variables were evaluated:

- Gerota’s fascia thickness (mm);
- Distance from tumor to renal sinus (mm);
- Contact surface area (CSA, cm^2^);
- Tumor depth (mm);
- Tumor radius (mm);
- Nearness to collecting system (mm);
- Renal diameters: anteroposterior, laterolateral, and longitudinal (mm);
- Tumor diameters: anteroposterior, laterolateral, and longitudinal (mm);
- Medullary invasion (yes/no);
- Hilar location (yes/no);
- Tumor margin (linear/irregular);
- Presence of pseudocapsule (yes/no);
- Presence of necrosis (yes/no);
- Tumor nature (solid/cystic);
- Tumor rim location (medial/lateral);
- Location relative to polar lines (upper/mid/lower);
- Longitudinal location (superior/midline/inferior);
- Tumor location (anterior/posterior);
- Exophytic rate (>50%/<50%/endophytic);
- RENAL nephrometry score (4–6/7–9/10–12).

Preoperative multidetector CT included non-contrast, arterial, venous, and excretory phases with 1–3 mm slice thickness. CSA was obtained by manual contouring of the tumor–parenchyma interface on consecutive venous-phase axial slices, with cross-checks on arterial and excretory phases to minimize artifacts; the three-dimensional surface was then computed with a vendor workstation (e.g., GE AW Server). CSA was expressed in cm^2^. Gerota’s fascia thickness was measured orthogonally from the renal capsule to the inner margin of the fascia at the site of maximal tumor bulging, expressed in millimeters.

All measurements were independently performed by four board-certified genitourinary radiologists, blinded to intraoperative and postoperative data, and following a standardized manual of operations. Inter-reader agreement for CSA and fascia thickness was quantified using the intraclass correlation coefficient (ICC, two-way random effects, absolute agreement). We report ICC (2,1) for single measurements and ICC (2,k) for the average of four readers.

### 2.3. Baseline, Perioperative and Functional Outcomes

Baseline clinical data included age, body mass index (BMI), sex, comorbidities (hypertension, diabetes), and ASA score (grouped as 1–2 vs. 3–4). Tumor side (right/left), surgical approach (laparoscopic vs. robotic), preoperative estimated glomerular filtration rate (eGFR, mL/min/1.73 m^2^, CKD-EPI formula), preoperative hemoglobin (Hb), and chronic kidney disease (CKD) stage were also collected. Tumor complexity was assessed using the RENAL score and the 2017 TNM clinical stage.

Perioperative outcomes included estimated blood loss (EBL), hemoglobin at discharge, ΔHb from baseline, hospital length of stay (LOS), and complications per Clavien–Dindo classification (with major complications defined as grade ≥ 3). Pathology data included pT stage and histologic subtype (benign vs. malignant).

Renal function was reassessed postoperatively with eGFR at discharge and at last follow-up. Acute kidney injury (AKI) was defined as an eGFR drop > 50% from baseline. Severe CKD upstaging was defined as progression to CKD stage ≥ 3b. End-stage renal disease (ESRD) was defined as new-onset CKD stage 4–5 at any time point.

A composite Trifecta was defined by achievement of:

- Negative surgical margins;
- Absence of major complications (Clavien–Dindo ≥ 3);
- eGFR decline < 30% at discharge.

The primary aim of this study was to identify specific preoperative CT-derived radiologic variables independently associated with the decision to perform renorrhaphy versus adopt a sutureless strategy during off-clamp partial nephrectomy. As a secondary objective, we compared perioperative and functional outcomes between cohorts stratified by reconstructive technique, to assess the clinical safety and efficacy of both approaches in a real-world dual-institutional setting.

### 2.4. Statistical Analysis

Continuous variables were expressed as medians with interquartile ranges (IQR), while categorical variables were reported as absolute numbers and percentages. Associations between radiologic variables and reconstructive strategy (renorrhaphy vs. sutureless) were assessed using univariable logistic regression. Variables with statistical significance (*p* < 0.05) in univariable testing were included in multivariable logistic regression to identify independent predictors. The primary modelling strategy was a multivariable logistic regression pre-specifying anatomically grounded predictors (contact surface area, tumor radius, Gerota’s fascia thickness) and adjusting for clinical tumor size. In view of the sample size and number of outcome events, mixed-effects models with centre/surgeon terms and propensity-weighted approaches were not pursued to avoid model instability and overfitting. Effect estimates were reported with 95% confidence intervals, and inferences were considered exploratory and hypothesis-generating.

Logistic regression analyses were conducted with CSA treated both as a dichotomous and as a continuous variable. The 15 cm^2^ threshold was adopted a priori from prior series, while restricted cubic splines were used to account for potential non-linear effects. Sensitivity analyses evaluated alternative cut-offs (12 cm^2^ and 18 cm^2^). Model performance was quantified using the C-statistic, calibration plots, and Brier score, with exploratory decision-curve analysis to assess potential clinical utility.

Multicollinearity among morphometric predictors (CSA, radius, depth, nearness) was examined through variance inflation factors and pairwise correlations; penalized regression was additionally applied as a robustness check.

Renal function over time was analyzed using linear mixed-effects models adjusted for baseline eGFR, accounting for repeated measures within patients. To enhance transparency, all effect estimates were reported with 95% confidence intervals, and instances of *p* = 0.000 were uniformly expressed as *p* < 0.001.

## 3. Results

From the original cohort of 219 patients, 18 were excluded (12 due to inadequate follow-up and 6 for incomplete baseline information), leaving 201 evaluable cases for the final analysis. The selection process is depicted in Figure 1. A total of 201 patients undergoing off-clamp partial nephrectomy were included, with 101 (50.2%) treated using a sutureless technique and 100 (49.8%) undergoing conventional renorrhaphy. More specifically, the sutureless technique was adopted in 39 of 111 cases (35.1%) at Regina Elena and in 23 of 90 cases (25.6%) at Campus Bio-Medico.

Baseline demographic and clinical characteristics were comparable between the two groups (Table 1). No significant differences were observed in age, sex, BMI, comorbidities (diabetes, hypertension), ASA score, surgical approach, tumor side, preoperative eGFR, or hemoglobin levels (each *p* > 0.1). Tumor size was slightly smaller in the sutureless group (3.1 vs. 3.6 cm; *p* = 0.04), while RENAL scores and clinical T stages were evenly distributed. Preoperative CKD stages also showed a similar distribution between groups (stages 1–2–3a: 42–50–9 vs. 39–52–9; *p* = 0.95).

Radiologic and morphometric features are detailed in Supplementary Table S1. Tumors in the renorrhaphy group had larger contact surface areas (CSA > 15 cm^2^: 76.0% vs. 56.4%; *p* = 0.002) and a greater median tumor radius (18.0 vs. 15.3 mm; *p* = 0.01). Gerota’s fascia was significantly thinner in the sutureless cohort (71.3% vs. 54.0% with <10 mm; *p* = 0.01). No significant differences were observed in tumor depth, sinus distance, proximity to the collecting system, renal diameters, or tumor location parameters (each *p* > 0.3). The ICC (2,1) for CSA was 0.91 (95% CI 0.86–0.95; n = 80), and for Gerota’s fascia thickness was 0.88 (95% CI 0.82–0.93; n = 80). Corresponding ICC (2,4) values were 0.96 and 0.94, respectively, indicating excellent agreement among readers.

Perioperative data are summarized in Table 2. Blood loss, hemoglobin drop, transfusion rates, and length of stay were comparable between groups (each *p* > 0.4). Clavien–Dindo grade ≥ 3 complications occurred in one patient (1.0%) per group: one urinoma requiring drainage in the sutureless cohort and one urinary leakage treated with stenting in the renorrhaphy group. Minor complications (Clavien I–II) were slightly more frequent in the renorrhaphy group, though without statistical significance (9.0% vs. 5.0%; *p* = 0.45). Positive surgical margin (PSM) rates were low in both groups (1.0% vs. 2.0%; *p* = 0.38). No significant differences were observed in postoperative eGFR or acute kidney injury rates (each *p* > 0.7).

Functional outcomes at a median follow-up of 26 months (IQR 18–34) are presented in Table 3. eGFR at last follow-up was comparable between groups (60.2 vs. 56.3 mL/min/1.73 m^2^; *p* = 0.21), with no significant differences in CKD stage distribution (stage ≥ 3b: 5.0% vs. 2.0%; *p* = 0.28). New-onset ESRD occurred in a single patient (1.0% vs. 0%). Trifecta achievement was high in both cohorts (92.1% vs. 89.0%; *p* = 0.41).

Predictors of renorrhaphy were assessed by logistic regression (Table 4 and Table 5). In the full model, CSA > 15 cm^2^ remained independently associated with renorrhaphy (OR 5.48; 95% CI 2.38–12.6; *p* < 0.001), while thinner Gerota’s fascia was inversely associated but not significant (OR 0.39; 95% CI 0.10–1.39; *p* = 0.14). With inclusion of tumor radius, CSA (OR 3.93; 95% CI 1.26–12.26; *p* = 0.02) and fascia thickness < 10 mm (OR 0.08; 95% CI 0.01–0.70; *p* = 0.02) reached significance as independent predictors of renorrhaphy after off-clamp partial nephrectomy. This integrated model improved predictive accuracy, with higher discrimination (ΔAUC 0.06) and significant reclassification metrics (NRI 0.14, *p* = 0.03; IDI 0.07, *p* = 0.04).

Sensitivity analyses stratified by surgical modality (robotic vs. laparoscopic), platform (da Vinci vs. Hugo), and tumor location (hilar vs. non-hilar; exophytic vs. endophytic) confirmed the stability of CSA, tumor radius, and fascia thickness as predictors, with no interaction effects detected (each *p* > 0.2). Both model specifications were retained to enhance transparency and highlight the robustness of results under varying levels of adjustment. Other clinical and morphometric parameters—including tumor depth, proximity to the collecting system, renal diameters, and longitudinal orientation—were not independently associated with renorrhaphy (each *p* > 0.1).

The final model demonstrated strong discrimination (AUC 0.81), satisfactory calibration, and a Brier score of 0.17. Analyses with CSA treated as a continuous variable, including restricted cubic splines and alternative thresholds (12 cm^2^ and 18 cm^2^), consistently confirmed its predictive role. Variance inflation factors were <2.5 for all predictors, excluding relevant collinearity, while penalized regression retained the same variables (CSA, radius, fascia thickness).

In linear mixed-effects modeling adjusted for baseline eGFR, no significant interaction was found between time and reconstructive technique (estimate −1.3 mL/min/1.73 m^2^; 95% CI −4.9 to 2.3; *p* = 0.46). At the last follow-up, no local recurrences were observed, while distant metastases occurred in three patients (1.5%), with no between-group difference (*p* = 0.24).

## 4. Discussion

In recent years, the reconstructive phase of minimally invasive partial nephrectomy (MIPN) has received renewed scrutiny, particularly concerning whether renorrhaphy is invariably necessary after tumor enucleation [7,20,21,22]. Traditionally regarded as the mainstay to ensure hemostasis and restore parenchymal continuity, renorrhaphy has gradually been re-evaluated in light of evidence linking it to secondary parenchymal damage, ischemia, occurrence of vascular complications such as pseudoaneurysms or arteriovenous fistulas, and impaired functional recovery [23,24,25]. Our group has previously described sutureless techniques during MIPN as a viable alternative when resection planes are favorable and bleeding is controllable with energy-based modalities [7]. Within this evolving surgical landscape, we aimed to determine which preoperative radiologic features could reliably predict the need for renorrhaphy, using a dual-institution cohort operated on by two experienced surgical teams, each applying a standardized off-clamp enucleative technique.

Our study revealed interesting findings. Among the baseline characteristics, clinical tumor size emerged as the only distinguishing feature between the two cohorts, being significantly greater in cases ultimately reconstructed with sutures (3.6 vs. 3.1 cm; *p* = 0.04). We subsequently observed through targeted imaging analysis that three radiologic variables demonstrated predictive potential. Contact surface area (CSA) >15 cm^2^ was significantly more prevalent in the renorrhaphy group (76.0% vs. 56.4%; *p* = 0.002), as was greater tumor radius (median 18.0 vs. 15.3 mm; *p* = 0.01). Conversely, a thin Gerota’s fascia (<10 mm) was more common among sutureless cases (71.3% vs. 54.0%; *p* = 0.01). These variables carry clear surgical reliability. A broader tumor–parenchyma interface and a larger radius are typically associated with a more extensive raw surface after enucleation, increasing the likelihood that mechanical reinforcement may be required. In contrast, a thinner perinephric wrapping can facilitate dissection and exposure, potentially allowing meticulous hemostasis without parenchymal compression [26,27].

Despite these anatomical features, perioperative outcomes were comparable across the reconstructive strategies. Key variables such as estimated blood loss, hemoglobin drop, length of stay, and complication rates did not differ significantly (each *p* ≥ 0.18). Notably, grade ≥ 3 complications were infrequent and reflected reconstructive nuances, with urinoma drainage observed after sutureless procedures and temporary stenting following renorrhaphy (*p* = 0.45). Early renal function outcomes mirrored this pattern of equivalence. Final eGFR values, CKD stage progression, and incidence of ESRD did not diverge meaningfully between groups (each *p* ≥ 0.28). These results should be interpreted with caution, as the observational design and low event frequency preclude formal equivalence or non-inferiority testing; nonetheless, when preoperative anatomy is favorable, the omission of sutures appears not to compromise surgical safety or functional preservation [22,28]. Moreover, the analysis was not designed or powered for oncologic endpoints; recurrence data are reported descriptively to provide context but should not be interpreted as evidence of oncologic equivalence.

The regression analysis further validated the independent contribution of specific imaging parameters to reconstructive decision-making. CSA > 15 cm^2^ remained a robust predictor of renorrhaphy in both the base (OR 5.48, 95% CI 2.38–12.60; *p* < 0.001) and expanded models (OR 3.93, 95% CI 1.26–12.26; *p* = 0.02). Tumor radius (OR 1.14 per mm, 95% CI 1.01–1.29; *p* = 0.04) and Gerota’s fascia <10 mm (OR 0.08 vs. >10 mm, 95% CI 0.01–0.70; *p* = 0.02) also retained significance. Gerota’s fascia < 10 mm showed borderline influence depending on model specification: in the base model including only radiologic variables it was not statistically significant (OR 0.39, *p* = 0.14), whereas in the expanded model adjusted for RENAL score descriptors it emerged as a significant predictor (OR 0.08, *p* = 0.02). This apparent discrepancy reflects the interplay between collinear morphometric variables: once RENAL components were introduced, the independent contribution of perinephric fascia became more clearly discernible. For transparency and to avoid over-simplification, both models were retained. While the expanded specification was considered the primary one, given its alignment with contemporary clinical practice where RENAL scoring is commonly applied, the parallel presentation allows readers to appreciate the robustness of CSA and radius and the conditional role of fascia thickness. Beyond statistical significance, the model demonstrated solid predictive performance, with high discrimination and adequate calibration. The consistency across alternative CSA thresholds and analytic specifications reinforces the clinical reliability of the predictors. These robustness checks mitigate concerns of collinearity and strengthen the generalizability of our findings.

Meaningfully, parameters such as tumor depth and nearness to the collecting system, although significant in the univariable analyses (each *p* ≤ 0.001), lost predictive value once the dominant effects of CSA and radius were considered, highlighting the interplay between linear proximity and overall parenchymal involvement. Importantly, the distribution of sutureless procedures across the two participating centers indicates that the association observed for reconstructive technique was not confined to a single institutional setting but consistently reproduced in distinct surgical environments. From a statistical perspective, these results underline the strength of our model despite the inherent limitations of a retrospective design. The approach was refined by employing mixed-effects modeling for renal function trajectories and by reporting effect sizes with confidence intervals, thereby enhancing robustness and interpretability. The predictors identified were not only statistically significant but also clinically grounded, and the consistency across institutions and platforms supports their generalizability. Additional stratified analyses reinforced this consistency, showing no heterogeneity of predictive associations when stratified by surgical modality, robotic platform, or tumor location. These findings suggest that the predictive framework is not contingent on technical factors or tumor topography but rather reflects intrinsic anatomic features. However, the absence of a propensity-matched analysis limits definitive assertions about equivalence between sutureless and renorrhaphy approaches. It is also important to recognize that the model predicts surgical judgment rather than an absolute gold standard of reconstructive necessity. Yet, this alignment with real-world decision-making is not a weakness but a reflection of clinical practice: during off-clamp tumor enucleation, the decision to suture or not invariably rests on intraoperative appraisal of bleeding control and parenchymal exposure. By isolating preoperative imaging features that anticipate these intraoperative judgments, the model provides a pragmatic tool to enhance planning, while not supplanting surgical expertise.

In both participating centers, partial nephrectomy has historically been carried out without hilar clamping, and off-clamp resection has long represented routine practice. This approach explains why our dataset exclusively reflects off-clamp procedures. On the one hand, this restriction provided the opportunity to eliminate ischemia as a confounder and to isolate the functional role of reconstruction and anatomical predictors. On the other hand, because the predictors identified are anatomical in nature, they are likely to retain clinical relevance even in clamped procedures. Nevertheless, our findings should be interpreted within the specific framework of off-clamp surgery, acknowledging that further validation in broader populations will be required. Moreover, the number of clinically relevant events—such as high-grade complications or renal deterioration—was low, potentially introducing a ceiling effect that obscures more nuanced differences. In particular, the very low rate of major complications (Clavien–Dindo ≥ 3) limited the statistical power to discern true differences in rare but serious adverse outcomes between reconstructive strategies. Consequently, the lack of statistical separation in this domain should be interpreted with caution and regarded as hypothesis-generating rather than confirmatory. Another key limitation is potential confounding by indication. The reconstructive method reflects real-time intraoperative judgment and may partly capture surgeon preference in addition to anatomy. Two experienced teams across two institutions contributed both sutureless and sutured cases within a uniform off-clamp enucleative workflow, which mitigates—though does not eliminate—this source of bias. Accordingly, the model should be interpreted as predicting the use of renorrhaphy rather than the absolute need for sutures. Future work should test these radiologic signatures in prospective designs using centre-level adjustment or balancing techniques to further address selection effects. In addition, while propensity-matched or weighted analyses could have provided more balanced cohorts, the limited sample size and low event frequency in our dataset precluded their reliable application. We therefore relied on parsimonious multivariable modeling, recognizing that propensity-based approaches should be pursued in larger collaborative series to further validate the present results.

Other limitations should be mentioned, such as the retrospective design and lack of balance across cohorts expose the study to potential selection biases, though attrition bias was mitigated by the completeness of follow-up in all included cases. Moreover, although radiologic assessments were centralized and reviewed by two operators, some degree of inter-observer variability cannot be excluded, particularly in parameters requiring manual measurement or qualitative interpretation.

Nonetheless, the study offers several strengths that help counterbalance these constraints. The consistency of the surgical technique, the centralized radiologic review, and the granular analysis of imaging features all contribute to a high level of methodological rigor. Furthermore, our findings underscore the value of integrating imaging signatures with clinical reasoning to guide intraoperative choices [29]. In this regard, the synergy between radiologists and surgeons is paramount. A detailed review of preoperative imaging, including three-dimensional reconstructions and morphometric analysis, may enhance both surgical planning and intraoperative adaptability, particularly in cases where the reconstructive strategy is not immediately apparent. These observations also inform the broader discourse surrounding nephrometry. While the R.E.N.A.L. score remains a valuable metric for grading anatomical complexity and predicting complications, it may fall short in informing reconstructive choices [11,30]. In our analysis, integration of CSA, tumor radius, and Gerota’s fascia thickness significantly improved discrimination beyond RENAL alone, underscoring the complementary value of this CT-based triad. Such markers may enhance the discriminatory power of established nephrometry and refine its role in preoperative planning. While the predictors identified here could theoretically be integrated into simplified risk scores or nomograms, the present series was not intended nor adequately powered for tool development or internal validation. Future collaborative efforts based on larger multicenter datasets will be essential to translate these radiologic signatures into practical decision-support instruments with validated probability thresholds.

Taken together, the evidence points toward a refined preoperative framework where radiologic precision informs tailored surgical strategy. Future models incorporating radiomics and machine-learning algorithms may further expand this approach, offering real-time, anatomy-specific guidance. Although such tools are not substitutes for surgical expertise, they may provide an additional layer of insight when deciding among technical options during MIPN.

## 5. Conclusions

In the context of minimally invasive, off-clamp partial nephrectomy, this dual-institutional study identifies a triad of CT-derived features—contact surface area, tumor radius, and perinephric fascia thickness—as independent predictors of reconstructive strategy. When morphologic conditions are favorable, renorrhaphy can often be safely omitted without statistically significant differences in perioperative safety or long-term functional preservation. These findings support a shift toward morphology-driven surgical planning and suggest that established nephrometry systems may be strengthened by integrating additional radiologic variables. Looking forward, the quantitative nature of these markers offers an opportunity for AI-based modeling to refine preoperative stratification and improve decision-making in nephron-sparing surgery.

## Figures and Tables

**Table 1:** Baseline data.

Variable (*n*, %)	Sutureless n = 101 (50.2%)	Renorrhaphy n = 100 (49.8%)	*p*-Value
**Age (years, median [IQR])**	64 [58–70]	65 [59–71]	0.65
**BMI (kg/m^2^, median [IQR])**	26.4 [24.2–29.3]	26.9 [24.8–30.1]	0.48
**ASA score (n, %)** **1–2** **3–4**	60 (59.4%) 41 (40.6%)	54 (54%) 46 (46%)	0.59
**Gender (n,%)** **Male** **Female**	62 (61.4%) 39 (38.6%)	64 (64%) 36 (36%)	0.72
**Diabetes (n,%)**	18 (17.8%)	20 (20%)	0.71
**Hypertension (n,%)**	57 (56.4%)	62 (62%)	0.42
**Surgical approach (n,%)** **Laparoscopic** **Robotic**	12 (11.9%) 89 (88.1%)	13 (13%) 87 (87%)	0.84
**Tumor side (n, %)** **Right** **Left**	52 (51.5%) 49 (48.5%)	50 (50%) 50 (50%)	0.66
**Clinical tumor size (cm, median [IQR])**	3.1 [2.4–4.3]	3.6 [2.6–4.8]	**0.04**
**Preoperative eGFR (mL/min/1.73 m^2^, median [IQR])**	82 [72–91]	78 [68–88]	0.06
**Preoperative hemoglobin (g/dL, median [IQR])**	13.8 [12.9–14.7]	13.6 [12.8–14.5]	0.21
**R.E.N.A.L. score (median [IQR])**	7 [6–9]	7 [6–9]	0.88
**R.E.N.A.L. score (n, %)** **4–6 (low complexity)** **7–9 (moderate complexity)** **10–12 (high complexity)**	25 (24.8%) 66 (65.3%) 10 (9.9%)	24 (24%) 66 (66%) 10 (10%)	0.94
**Clinical T stage (n, %)** **cT1a** **cT1b** **cT2**	62 (61.4%) 35 (34.7%) 4 (4%)	59 (59%) 36 (36%) 5 (5%)	0.73
**Preoperative CKD stage (n,%)** **1** **2** **3a**	42 (41.6%) 50 (49.5%) 9 (8.9%)	39 (39%) 52 (52%) 9 (9%)	0.95

BMI: body mass index; IQR: interquartile range; CKD: chronic kidney disease.

**Table 2:** Perioperative data.

Variable (n, %)	Sutureless n = 101 (50.2%)	Renorrhaphy n = 100 (49.8%)	*p*-Value
**Operative time**	65 (55–80)	65 (50–80)	0.80
**Estimated blood loss (mL, median, IQR)**	100 (60–180)	105 (60–190)	0.69
**Hb at discharge (g/dL, median, IQR)**	11.9 (10.9–12.8)	11.7 (10.7–12.7)	0.42
**ΔHb (g/dL, median, IQR)**	−2.1 (−1.1; −3.1)	−2.2 (−1.2; −3.2)	0.61
**Perioperative complications (Clavien–Dindo, n, %)** **Clavien I-II** **Grade ≥ 3**	5 (5.0%) fever (n = 2), transient ileus (n = 1), transfusion (n = 2) 1 (1.0%) urinoma requiring percutaneous drainage	9 (9.0%) transfusion (n = 4), fever (n = 2), anemia requiring iron (n = 3) 1 (1.0%)urinary leakage requiring double-J stenting	0.45
**PSM (n, %)**	1 (1.0%)	2 (2.0%)	0.38
**LOS (days, median, IQR)**	4 (3–5)	4 (3–5)	0.83
**eGFR at discharge (mL/min/1.73 m^2^, median, IQR)**	52.3 (43.1–63.7)	51.5 (42.0–62.2)	0.71
**ΔeGFR (median, IQR)**	−9.3 (IQR −5.1 to −22.4; 95% CI −11.2 to −7.4)	−9.7 (IQR −5.4 to −23.1; 95% CI −11.6 to −7.8)	0.74
**AKI (ΔeGFR >50%, n, %)**	1 (1.0%)	4 (4.0%)	0.18
**pT stage (n, %)** **pT1a** **pT1b** **pT2-pT3a**	65 (64.4%) 23 (22.8%) 13 (12.9%)	60 (60%) 25 (25%) 15 (15%)	0.11
**Histology (n,%)** **Benign** **RCC or other malignant**	18 (17.8%) 83 (82.2%)	14 (14%) 86 (86%)	0.26

Hb: hemoglobin; PSM: positive surgical margin; LOS: length of stay; eGFR: estimated glomerular filtration rate; AKI: acute kidney injury; RCC: renal cell carcinoma.

**Table 3:** Functional outcomes.

Variable	Overall Cohort (n = 201)	Renorrhaphy (n = 100)	Sutureless (n = 101)	*p*-Value
**Follow-up (months, median, IQR)**	26 (18–34)	25 (18–33)	27 (19–35)	0.48
**eGFR at last follow-up (mL/min/1.73 m^2^)**	58.1 (47.6–69.4)	56.3 (45.8–67.9)	60.2 (49.2–70.8)	0.21
**CKD stage at last follow-up (n, %)** **1** **2** **3a** **3b** **4** **5**	28 (13.9%) 49 (24.4%) 105 (52.2%) 15 (7.5%) 3 (1.5%) 1 (0.5%)	12 (12%) 22 (22%) 53 (53%) 9 (9%) 3 (3%) 1 (1%)	16 (15.8%) 27 (26.7%) 52 (51.5%) 6 (5.9%) - -	0.43
**Severe CKD upstaging (≥stage 3b), n (%)**	7 (3.5%)	5 (5.0%)	2 (2.0%)	0.28
**Newly onset ESRD (n, %)**	1 (0.5%)	1 (1.0%)	0 (0.0%)	0.32
**Trifecta achievement (n, %)**	182 (90.5%)	89 (89.0%)	93 (92.1%)	0.41

eGFR: estimated glomerular filtration rate; CKD: chronic kidney disease; ESRD: end-stage renal disease.

**Table 4:** Univariable and multivariable logistic regression identifying predictors of renorrhaphy after off-clamp partial nephrectomy.

Variable	Univariable Analysis				Multivariable Analysis			
	OR	95.0% CI			OR	95.0% CI		
		Lower	Higher	*p*-Value		Lower	Higher	*p*-Value
**Gerota thickness (<10 mm vs. >10 mm)**	0.24	0.08	0.69	**0.008**	0.39	0.10	1.39	0.14
**Contact surface area (>15 cm^2^ vs. <15 cm^2^)**	4.77	2.47	9.21	**<0.001**	5.48	2.38	12.6	**<0.001**
**R.E.N.A.L. score** **(4–6 vs. 7–9** **4–6 vs. 10–12)**	0.79 2.16	0.40 0.95	1.54 4.89	0.49 0.06	- -	- -	- -	- -
**Renal antero-posterior diameter (mm)**	1.01	0.98	1.04	0.266	-	-	-	-
**Renal latero-lateral diameter (mm)**	1.03	0.99	1.06	0.07	-	-	-	-
**Renal length superior-inferior diameter (mm)**	1.02	0.99	1.04	0.08	-	-	-	-
**Medullary invasion (no vs. yes)**	2.02	0.63	6.43	0.233	-	-	-	-
**Margin (linear vs. irregular)**	1.20	0.57	2.52	0.616	-	-	-	-
**Tumor pseudocapsule (no vs. yes)**	1.40	0.71	2.77	0.325	-	-	-	-
**Necrosis (no vs. yes)**	1.36	0.68	2.73	0.378	-	-	-	-
**Nature of the tumour (cystic vs. solid)**	1.87	0.92	3.80	0.08	-	-	-	-

**Table 5:** Regression model incorporating RENAL score variables for prediction of renorrhaphy in off-clamp partial nephrectomy.

Variable	Univariable Analysis				Multivariable Analysis			
	OR	95.0% CI			OR	95.0% CI		
		Lower	Higher	*p*-Value		Lower	Higher	*p*-Value
**Gerota thickness (<10 mm vs. >10 mm)**	0.24	0.08	0.69	**0.008**	0.08	0.01	0.70	**0.02**
**Distance from mass to renal sinus (mm)**	0.94	0.90	0.99	**0.032**	1.02	0.92	1.14	0.66
**Contact surface area (>15 cm^2^ vs. <15 cm^2^)**	4.77	2.47	9.21	**<0.001**	3.93	1.26	12.26	**0.02**
**Tumor depth (mm)**	1.06	1.03	1.09	**<0.001**	1.04	0.96	1.12	0.36
**Tumor radius (mm)**	1.05	1.01	1.08	**0.002**	1.14	1.01	1.29	**0.04**
**Nearness to collecting system (mm)**	0.86	0.78	0.94	**0.001**	0.92	0.78	1.08	0.30
**Antero-posterior diameter renal mass (mm)**	1.02	1.008	1.043	**0.003**	0.99	0.92	1.08	0.93
**Latero-lateral diameter renal mass (mm)**	1.02	1.008	1.043	**0.004**	1.03	0.94	1.13	0.54
**R.E.N.A.L. score** **(4–6 vs. 7–9** **4–6 vs. 10–12)**	0.79 2.16	0.40 0.95	1.54 4.89	0.49 0.064	- -	- -	- -	- -
**Renal antero-posterior diameter (mm)**	1.01	0.98	1.04	0.266	-	-	-	-
**Renal latero-lateral diameter (mm)**	1.03	0.99	1.06	0.07	-	-	-	-
**Renal length superior-inferior diameter (mm)**	1.02	0.99	1.04	0.08	-	-	-	-
**Medullary invasion (no vs. yes)**	2.02	0.63	6.43	0.233	-	-	-	-
**Hilar mass (no vs. yes)**	1.56	0.65	3.75	0.313	-	-	-	-
**Margin (linear vs. Irregular)**	1.20	0.57	2.52	0.616	-	-	-	-
**Tumor pseudocapsule (no vs. yes)**	1.40	0.71	2.77	0.325	-	-	-	-
**Necrosis (no vs. yes)**	1.36	0.68	2.73	0.378	-	-	-	-
**Nature of the tumour (cystic vs. solid)**	1.87	0.92	3.80	0.08	-	-	-	-
**Renal rim (medial vs. lateral)**	0.72	0.38	1.35	0.307	-	-	-	-
**Location relative to polar line** **Upper vs. middle** **Upper vs. lower**	2.08 1.37	0.95 0.70	4.54 2.65	0.06 0.35	- -	- -	- -	- -
**Tumor location (anterior vs. posterior)**	0.72	0.40	1.32	0.301	-	-	-	-
**Longitudinal location** **Upper vs. middle** **Upper vs. lower**	0.60 0.60	0.29 0.29	1.24 1.25	0.17 0.17	- -	- -	- -	- -
**Exopythic rate** **>50 vs. < 50%** **>50% vs. endophytic**	1.18 1.65	0.64 0.51	2.17 5.30	0.59 0.39	- -	- -	- -	- -

## Data Availability

The data presented in this study are available on request from the corresponding author.

## Supplementary Materials

The following supporting information can be downloaded from https://www.mdpi.com/article/10.3390/cancers17193236/s1, Table S1: Radiologic and tumor-specific characteristics according to renorraphy versus sutureless technique.

## Abbreviations

The following abbreviations are used in this manuscript:

ocMIPN	Off-clamp minimally invasive partial nephrectomy
CSA	Contact surface area
MIPN	Minimally invasive partial nephrectomy
ICC	Intraclass correlation coefficient
BMI	Body mass index
eGFR	Estimated glomerular filtration rate
Hb	Preoperative hemoglobin
CKD	Chronic kidney disease
EBL	Estimated blood loss
LOS	Hospital length of stay
AKI	Acute kidney injury
ESRD	End-stage renal disease
IQR	Interquartile ranges
PSM	Positive surgical margin
RCC	Renal Cell Carcinoma

## References

[1] Hinata N., Murakami S., Nakano Y., Hara I., Kondo T., Hamamoto S., Shiroki R., Nagayama J., Kawakita M., Eto M. (2024). Efficacy of robot-assisted partial nephrectomy compared to conventional laparoscopic partial nephrectomy for completely endophytic renal tumor: A multicenter, prospective study. Int. J. Clin. Oncol..

[2] Mahmud H., Erlich T., Zilberman D.E., Rosenzweig B., Portnoy O., Dotan Z.A. (2025). Robotic partial nephrectomy is associated with a lower incidence of urine leakage following nephron-sparing surgery for kidney tumors compared to open and laparoscopic approaches. World J. Urol..

[3] Peyronnet B., Tondut L., Bernhard J.C., Vaessen C., Doumerc N., Sebe P., Pradere B., Guillonneau B., Khene Z.E., Nouhaud F.X. (2018). Impact of hospital volume and surgeon volume on robot-assisted partial nephrectomy outcomes: A multicentre study. BJU Int..

[4] Thakker P.U., O’Rourke T.K., Hemal A.K. (2023). Technologic advances in robot-assisted nephron sparing surgery: A narrative review. Transl. Androl. Urol..

[5] Juvet T.S., Thompson R.H., Potretzke A.M. (2020). Robot-assisted partial nephrectomy is safe and effective for complex renal masses when performed by experienced surgeons. Transl. Androl. Urol..

[6] Deng W., Li J., Liu X., Chen L., Liu W., Zhou X., Zhu J., Fu B., Wang G. (2020). Robot-assisted versus laparoscopic partial nephrectomy for anatomically complex T1b renal tumors with a RENAL nephrometry score ≥7: A propensity score-based analysis. Cancer Med..

[7] Brassetti A., Misuraca L., Anceschi U., Bove A.M., Costantini M., Ferriero M.C., Guaglianone S., Mastroianni R., Torregiani G., Covotta M. (2023). Sutureless Purely off-Clamp Robot-Assisted Partial Nephrectomy: Avoiding Renorrhaphy Does Not Jeopardize Surgical and Functional Outcomes. Cancers.

[8] Franco A., Riolo S., Tema G., Guidotti A., Brassetti A., Anceschi U., Bove A.M., D’Annunzio S., Ferriero M., Mastroianni R. (2024). Renal Function Preservation in Purely Off-Clamp Sutureless Robotic Partial Nephrectomy: Initial Experience and Technique. Diagnostics.

[9] Anceschi U., Ferriero M.C., Tuderti G., Brassetti A., Bertolo R., Capitanio U., Larcher A., Garisto J., Antonelli A., Mottrie A. (2021). Head to Head Impact of Margin, Ischemia, Complications, Score Versus a Novel Trifecta Score on Oncologic and Functional Outcomes After Robotic-assisted Partial Nephrectomy: Results of a Multicenter Series. Eur. Urol. Focus..

[10] Pandolfo S.D., Wu Z., Campi R., Bertolo R., Amparore D., Mari A., Verze P., Manfredi C., Franco A., Ditonno F. (2024). Outcomes and Techniques of Robotic-Assisted Partial Nephrectomy (RAPN) for Renal Hilar Masses: A Comprehensive Systematic Review. Cancers.

[11] Kutikov A., Uzzo R.G. (2009). The RENAL nephrometry score: A comprehensive standardized system for quantitating renal tumor size, location and depth. J. Urol..

[12] Ficarra V., Novara G., Secco S., Macchi V., Porzionato A., De Caro R., Artibani W. (2009). Preoperative aspects and dimensions used for an anatomical (PADUA) classification of renal tumours in patients who are candidates for nephron-sparing surgery. Eur. Urol..

[13] Li X.R., Li K.P., Zuo J.L., Yang W., Tan H., Wang W.Y., Chen S.Y., Ma J.H., Bao J.S., Yue Z.J. (2023). Perioperative, functional, and oncologic outcomes of minimally-invasive surgery for highly complex renal tumors (RENAL or PADUA score ≥ 10): An evidence-based analysis. J. Robot. Surg..

[14] Tuderti G., Mastroianni R., Anceschi U., Bove A.M., Brassetti A., Ferriero M., Misuraca L., Guaglianone S., Costantini M., Torregiani G. (2023). Assessing the Trade-off Between the Safety and Effectiveness of Off-clamp Robotic Partial Nephrectomy for Renal Masses with a High RENAL Score: A Propensity Score-matched Comparison of Perioperative and Functional Outcomes in a Multicenter Analysis. Eur. Urol. Focus..

[15] De Nunzio C., Tema G., Brassetti A., Anceschi U., Bove A.M., D’Annunzio S., Ferriero M., Mastroianni R., Misuraca L., Guaglianone S. (2024). Purely Off-Clamp Sutureless Robotic Partial Nephrectomy for Novice Robotic Surgeons: A Multi-Institutional Propensity Score-Matched Analysis. J. Clin. Med..

[16] Liu P., Li Y., Shi B., Zhang Q., Guo H. (2022). The Outcome of Sutureless in Partial Nephrectomy: A Systematic Review and Meta-Analysis. Biomed. Res. Int..

[17] Wu X., Zhou J., Chen W., Cai W., Liu D., Huang Y., Tricard T., Chen Y., Xue W. (2024). Retroperitoneoscopic Clampless, Sutureless Hybrid Therapy in the Management of Renal Hilar Tumors. Ann. Surg. Oncol..

[18] Rac G., Ellis J.L., Lanzotti N.J., McCormick M.E., Felice M.D., Janakiraman S., Desai S., Halgrimson W., Patel H.D., Gupta G.N. (2024). The evolution of tumor enucleation partial nephrectomy: A comparison of perioperative outcomes for sutureless hemostatic bandage as an alternative to standard renorrhaphy. J. Surg. Oncol..

[19] Schiavina R., Bianchi L., Borghesi M., Chessa F., Cercenelli L., Marcelli E., Brunocilla E. (2019). Three-dimensional digital reconstruction of renal model to guide preoperative planning of robot-assisted partial nephrectomy. Int. J. Urol..

[20] Giulioni C., Di Biase M., Marconi A., Sortino G., Diambrini M., Iacovelli V., Giannubilo W., Ferrara V. (2022). Clampless Laparoscopic Tumor Enucleation for Exophytic Masses Greater Than 4 cm: Is Renorrhaphy Necessary?. J. Laparoendosc. Adv. Surg. Tech. A.

[21] Ito H., Nakane K., Hagiwara N., Kawase M., Kato D., Iinuma K., Ishida K., Enomoto T., Nezasa M., Tobisawa Y. (2024). Impact of Robotic-Assisted Partial Nephrectomy with Single Layer versus Double Layer Renorrhaphy on Postoperative Renal Function. Curr. Oncol..

[22] Li W., Hua B., Song S., Pan W., Yang Q., Xu B. (2024). From sutureless to standard: A comprehensive analysis of conversion rates in laparoscopic partial nephrectomy. BMC Urol..

[23] Shatagopam K., Bahler C.D., Sundaram C.P. (2020). Renorrhaphy techniques and effect on renal function with robotic partial nephrectomy. World J. Urol..

[24] Kilic S., Ates M. (2025). Sutureless versus conventional suture renorrhaphy in clampless robotic partial nephrectomy: A single center propensity score matching analysis. Actas Urol. Esp..

[25] Tufano A., Asero V., Proietti F., Flammia R.S., Franco G., Leonardo C. (2022). Arteriovenous fistula after robotic partial nephrectomy: Case report and narrative review. Radiol. Case Rep..

[26] Anceschi U., Brassetti A., Tuderti G., Consiglia Ferriero M., Minervini A., Mari A., Grosso A.A., Carini M., Capitanio U., Larcher A. (2022). Risk factors for progression of chronic kidney disease after robotic partial nephrectomy in elderly patients: Results from a multi-institutional collaborative series. Minerva Urol. Nephrol..

[27] Sharma G., Shah M., Ahluwalia P., Dasgupta P., Challacombe B.J., Bhandari M., Ahlawat R., Rawal S., Buffi N.M., Sivaraman A. (2023). Development and Validation of a Nomogram Predicting Intraoperative Adverse Events During Robot-assisted Partial Nephrectomy. Eur. Urol. Focus..

[28] Subirá-Rios D., Trapero-Moreno D., Caño-Velasco J., González-García J., Moncada-Iribarren I., Aragón-Chamizo J., Fernández-Tamayo A., DEMiguel-Campos E., Subirá-Ríos J., Perez-Mañanes R. (2023). A new surgical technique for sutureless partial nephrectomy: Renal sutureless device. Minerva Urol. Nephrol..

[29] Amparore D., Sica M., Verri P., Piramide F., Checcucci E., De Cillis S., Piana A., Campobasso D., Burgio M., Cisero E. (2024). Computer Vision and Machine-Learning Techniques for Automatic 3D Virtual Images Overlapping During Augmented Reality Guided Robotic Partial Nephrectomy. Technol. Cancer Res. Treat..

[30] Hua J., Chen X. (2025). Accuracy of RENAL nephrometry score in predicting perioperative outcomes of minimally invasive partial nephrectomy: Impact of different surgical techniques. Transl. Androl. Urol..

