# Supplementary material for: Profiling the Kidney Before the Incision: CT-Derived Signatures Steering Reconstructive Strategy After Off-Clamp Minimally Invasive Partial Nephrectomy
Source: Cancers (Basel). 2025 Oct 5;17(19):3236. doi: 10.3390/cancers17193236 (PMC12523574; doi:10.3390/cancers17193236)
Supplement: Supplementary file 1 [file cancers-17-03236-s001.zip › cancers-3859336-supplementary.pdf]

<u>Table S1. Radiologic and tumor-specific characteristics according to renorraphy versus sutureless technique.</u>

Variable	Unit / Categories	Sutureless (n=101)	Renorraphy (n=100)	p-value
**Gerota thickness <10 mm**	n (%)	72 (71.3%)	54 (54.0%)	**0.01**
**Contact Surface Area >15 cm²**	n (%)	57 (56.4%)	76 (76.0%)	**0.002**
**Tumor radius**	mm, median (IQR)	15.3 (13.1–18.1)	18.0 (14.8–22.2)	0.01
**Tumor depth**	mm, median (IQR)	9.5 (7.0–11.6)	9.8 (6.7–11.9)	0.41
**Distance to renal sinus**	mm, median (IQR)	12.1 (9.5–15.6)	11.4 (8.3–16.1)	0.43
**Nearness to collecting system**	mm, median (IQR)	7.6 (4.4–10.8)	6.8 (3.9–10.5)	0.36
**CSA (continuous)**	cm², median (IQR)	14.6 (11.4–18.9)	17.3 (13.2–22.1)	**0.01**
**Renal size AP**	mm, median (IQR)	51.2 (47.5–55.0)	52.3 (46.9–55.7)	0.47
**Renal size LL**	mm, median (IQR)	55.4 (50.8–59.6)	56.0 (51.7–60.9)	0.59
**Renal size SI**	mm, median (IQR)	94.2 (88.0–101.1)	95.5 (90.2–102.3)	0.44
**Medullary invasion (yes)**	n (%)	7 (6.9%)	11 (11.0%)	0.34
**Hilar mass (yes)**	n (%)	12 (11.9%)	18 (18.0%)	0.23
**Margin: polycyclic**	n (%)	33 (32.7%)	36 (36.0%)	0.67
**Pseudocapsule (present)**	n (%)	65 (64.4%)	71 (71.0%)	0.33
**Nature: solid**	n (%)	73 (72.3%)	81 (81.0%)	0.18
**Renal rim: medial**	n (%)	39 (38.6%)	41 (41.0%)	0.74
**Polar location**	Upper / Middle / Lower	38 / 42 / 21	41 / 40 / 19	0.91
**Tumor location**	Anterior / Posterior	44 (43.6%) / 57 (56.4%)	39 (39.0%) / 61 (61.0%)	0.54
**Longitudinal location**	Upper / Middle / Lower	31 / 38 / 32	29 / 39 / 32	0.96
**Exophytic rate**	>50% / <50% / endophytic	66 / 25 / 10	61 / 27 / 12	0.78
